# Supplementary material for: Genome-Wide Characterization of the PIFs Family in Sweet Potato and Functional Identification of IbPIF3.1 under Drought and Fusarium Wilt Stresses
Source: Int J Mol Sci. 2023 Feb 17;24(4):4092. doi: 10.3390/ijms24044092 (PMC9965949; doi:10.3390/ijms24044092)
Supplement: Supplementary file 1 [file ijms-24-04092-s001.zip › ijms-2182922-supplementary.pdf]

Table S1. Identification of *PIF* genes in *I. batatas*, *I. trifida*, and *I. triloba*

Homologous gene in	Gene Name	Gene ID	Chr.	Gene Position	
				Start	End
I. batatas	*IbPIF1.1*	g49455	Chr12	19073417	19081441
	IbPIF1.2	g5235	Chr2	6804816	6808090
	IbPIF3.1	g44153	Chr11	19555486	19562243
	IbPIF3.2	g35474	Chr9	10168851	10173675
	IbPIF4	g54841	Chr13	26280156	26288826
	IbPIF8	g9143	Chr2	35946300	35950799
I. triloba	*ItbPIF1.1*	itb07g18930.t1	Chr7	23334370	23340785
	ItbPIF1.2	itb04g26140.t1	Chr4	30541436	30545240
	ItbPIF3.1	itb01g10970.t1	Chr1	9605666	9610936
	ItbPIF3.2	itb10g13850.t2	Chr10	20118588	20123423
	ItbPIF4	itb02g05380.t1	Chr2	3230198	3233573
	ItbPIF8	itb04g02660.t1	Chr4	1599998	1604800
I. trifida	*ItfPIF1.1*	itf07g16900.t1	Chr7	17046521	17053122
	ItfPIF1.2	itf04g26810.t1	Chr4	27399842	27403233
	ItfPIF3.1	itf01g17510.t1	Chr1	18817393	18822493
	ItfPIF3.2	itf10g13440.t1	Chr10	16054537	16059414
	ItfPIF4	itf02g09990.t1	Chr2	8811093	8820340
	ItfPIF8	itf04g02560.t1	Chr4	1362466	1367240

Table S2: Information of *PIFs* in different species

IbPIF1.1	Ib12g49455	*Ipomoea batatas*
IbPIF1.2	Ib02g5235	
IbPIF3.1	Ib11g44153	
IbPIF3.2	Ib09g35474	
IbPIF4	Ib13g54841	
IbPIF8	Ib02g9143	
ItbPIF1.1	Itb07g18930	*Ipomoea triloba*
ItbPIF1.2	Itb04g26140	
ItbPIF3.1	Itb01g10970	
ItbPIF3.2	Itb10g13850	
ItbPIF4	Itb02g05380	
ItbPIF8	Itb04g02660	
ItfPIF1.1	Itf07g16900	*Ipomoea trifida*
ItfPIF1.2	Itf04g26810	
ItfPIF3.1	Itf01g17510	
ItfPIF3.2	Itf10g13440	
ItfPIF4	Itf02g09990	
ItfPIF8	Itf04g02560	
AtPIF1	AT2G20180	*Arabidopsis thaliana*
AtPIF2	AT2G46970	
AtPIF3	AT1G09530	
AtPIF4	AT2G43010	
AtPIF5	AT3G59060	
AtPIF6	AT3G62090	
AtPIF7	AT5G61270	
AtPIF8	AT4G00050	
OsPIL11	Os12g0610200	*Oryza sativa*
OsPIL12	Os03g0639300	
OsPIL13	Os03g0782500	
OsPIL14	Os07g0143200	
OsPIL15	Os01g0286100	
OsPIL16	Os05g0139100	
CsPIF1	TEA006532	*Camellia sinensis*
CsPIF3a	TEA033210	
CsPIF3b	TEA007077	
CsPIF7a	TEA025875	
CsPIF7b	TEA011633	
CsPIF8a	TEA032260	
CsPIF8b	TEA023842	
SlPIF1a	Solyc09g063010	*Solanum lycopersicum*
SlPIF1b	Solyc06g008030	
SlPIF3	Solyc01g102300	
SlPIF4	Solyc07g043580	
SlPIF7a	Solyc03g115540	
SlPIF7b	Solyc06g069600	
SlPIF8a	Solyc01g090790	
SlPIF8b	Solyc10g018510	
VvPIF1	LOC100264869	*Vitis vinifera*
VvPIF3	LOC100247781	
VvPIF4	LOC100262490	
VvPIF7	LOC100262138	
MdPIF1	MDP0000289642	*Malus domestica*
MdPIF2	MDP0000205358	
MdPIF3	MDP0000290263	
MdPIF4	MDP0000198404	
MdPIF5	MDP0000254650	
MdPIF7	MDP0000319248	
MdPIF8	MDP0000439540	
StPIF1a	PGSC0003DMG400018950	*Solanum tuberosum*
StPIF1b	PGSC0003DMG400014705	
StPIF3	PGSC0003DMG400018280	
StPIF4	PGSC0003DMG401015926	
StPIF7a	PGSC0003DMG400024554	
StPIF7b	PGSC0003DMG400033087	
StPIF8	PGSC0003DMG400025976	
DcPIF3	DCAR_029297	*Daucus carota*
DcPIF7.1	DCAR_016590	
DcPIF7.2	DCAR_026020	
DcPIF8.1	DCAR_015869	
DcPIF8.2	DCAR_025094	

Table S3. Primers used in this study

Primer name	Primer sequence (5'-3')
qRT-*IbPIF1.1*-F	TCCACGGTTTGCTATGCCA
qRT-*IbPIF1.1*-R	AGCATGGGATCGGGTTGG
qRT-*IbPIF1.2*-F	CCCTCCAGAATTCAGGCATC
qRT-*IbPIF1.2*-R	CCATCTCGTTCATTGAAGGTGT
qRT-*IbPIF3.1*-F	CTGGAGTAGTACCTGAAGCATCTGG
qRT-*IbPIF3.1*-R	TTAGAACCACCTGTCTTATCCATCG
qRT-*IbPIF3.2*-F	GGGCATTTCATCCAAAACAGA
qRT-*IbPIF3.2*-R	GGGCATTTCATCCAAAACAGA
qRT-*IbPIF4*-F	CGAGATGCATACACGCAGCTTA
qRT-*IbPIF4*-R	CCTAGTTCATCACAGGCAGCC
qRT-*IbPIF8*-F	TTCCATGATGCCTGATCCTTTA
qRT-*IbPIF8*-R	GGGCTTGTTGAAACTGCTGG
qRT-*NtDREB1A*-F	CTTGGAGGTTACCCATCCCG
qRT-*NtDREB1A*-R	AATGTTTCGGCCGCTTCAAC
qRT-*NtDREB1*B-F	CCGCCGACGCTAAGGATATT
qRT-*NtDREB1B*-R	TGGCGTTTCAGGAGTAGTGC
qRT-*NtDREB1D*-F	GCTATTGCATTAAGGGGCCG
qRT-*NtDREB1D*-R	TGAATATCCTTGGCGTCGGT
qRT-*NtPOD*-F	TGCGATGGATCGCTACTCCT
qRT-*NtPOD*-R	ACACCAGGGCACACTTTCTC
qRT-*NtPR1a*-F	AACCTTTGACCTGGGACGAC
qRT-*NtPR1a*-R	GCACATCCAACACGAACCGA
qRT-*NtHSR201*-F	CAGCAGTCCTTTGGCGTTGTC
qRT-*NtHSR201*-R	GCTCAGTTTAGCCGCAGTTGTG
qRT-*NtHSR515*-F	TTGGGCAGAATAGATGGGTA
qRT-*NtHSR515*-R	TTTGGTGAAAGTCTTGGCTC
actin-F	AGCAGCATGAAGATTAAGGTTGTAGCAC
actin-R	TGGAAAATTAGAAGCACTTCCTGTGAAC
IbPIF3.1-ORF-F	ATGCCTCTCTCTGAGTTTTTGAAGT
IbPIF3.1-ORF-R	CTACACTTTCCTCTGAGATGATGTTTG

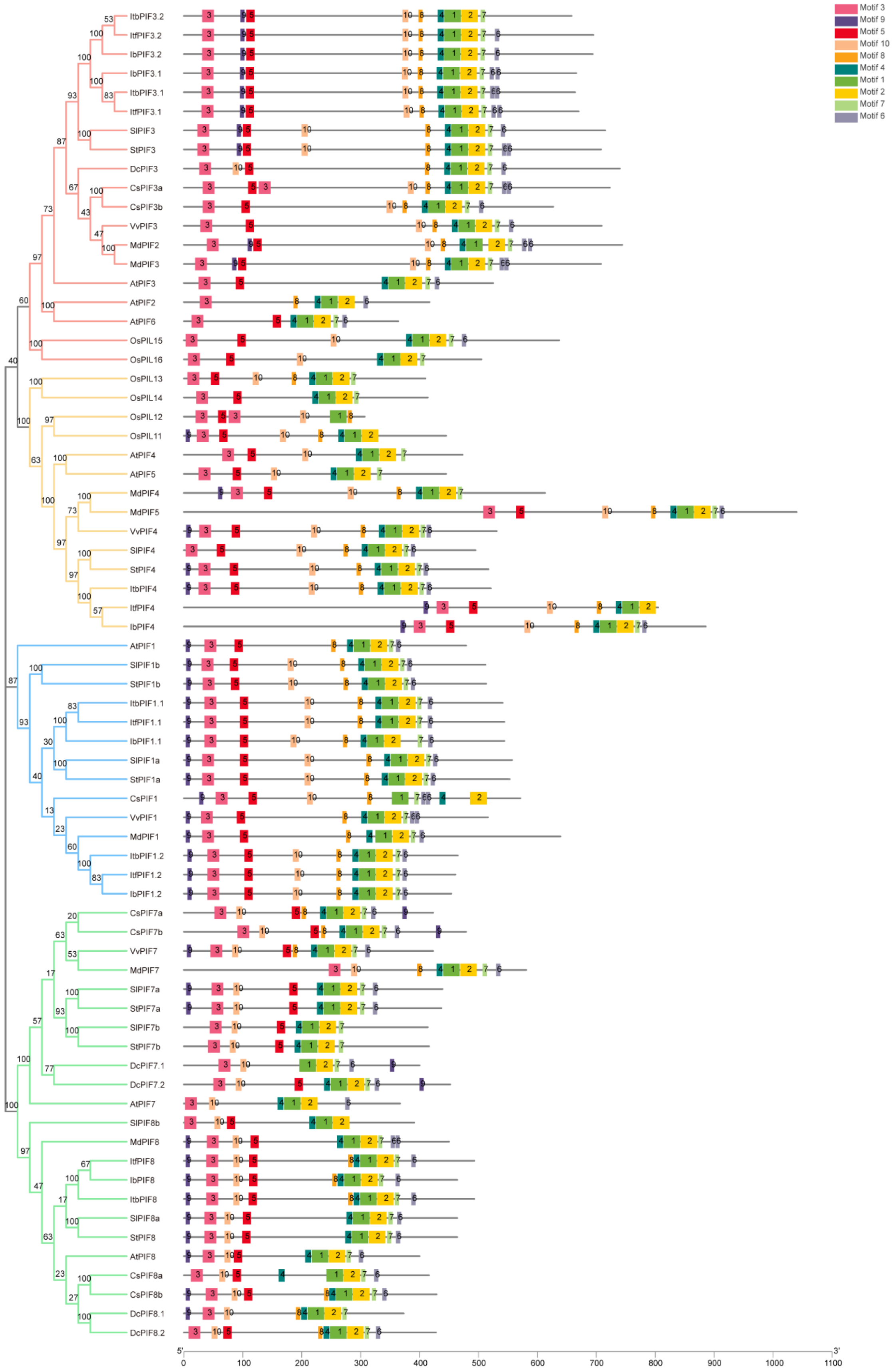

Figure S1. Conserved motifs analysis of PIFs in *A. thaliana, C. sinensis, D. carota, I. batatas, I.*

triloba, *I. trifida*, *M. domestica*, *O. sativa*, *S. lycopersicum*, *S. tuberosum*, and *V. vinifera*. The 10 conserved motifs were shown in different colors.

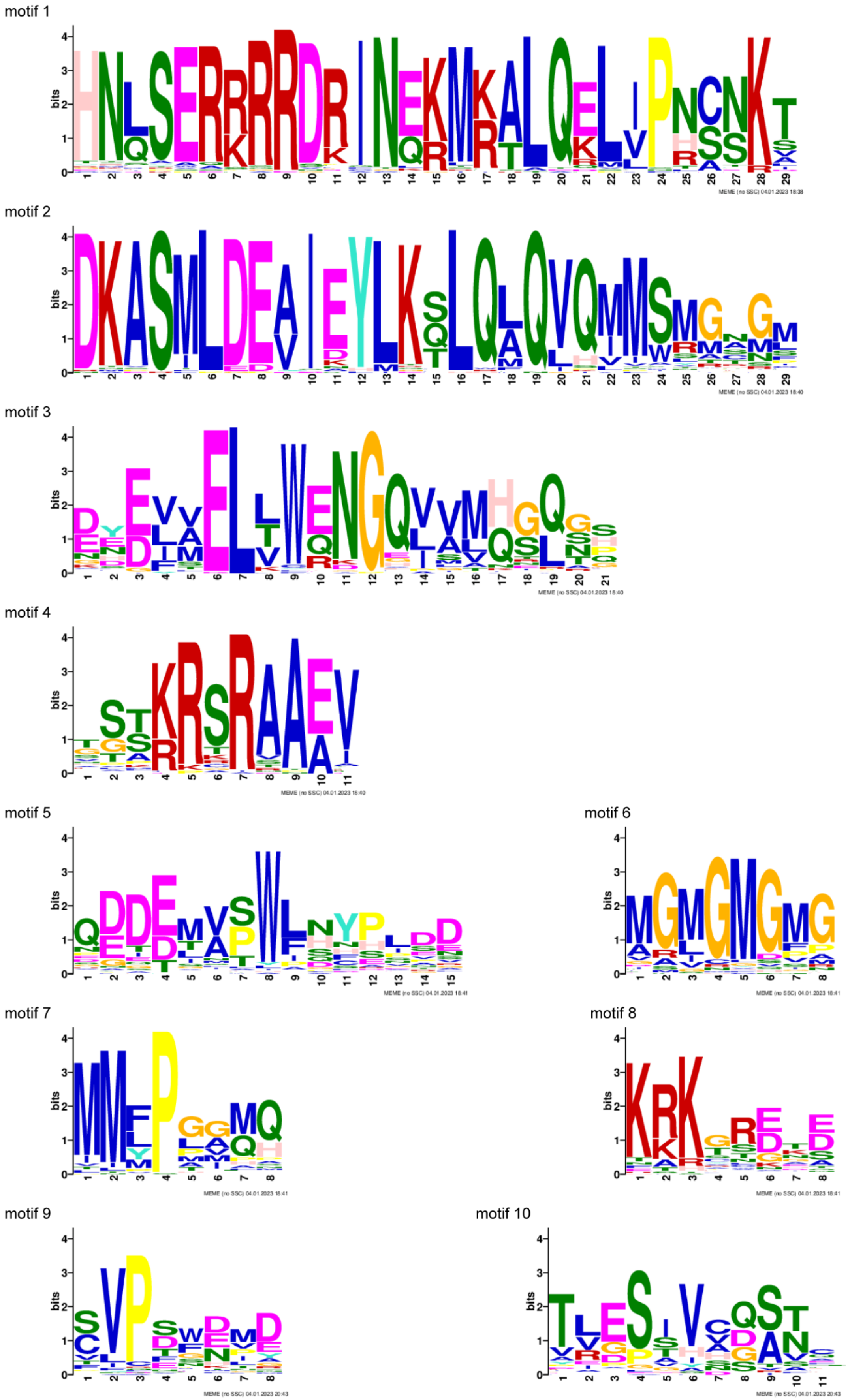

Figure S2. Sequence logos of the 10 conserved motifs.

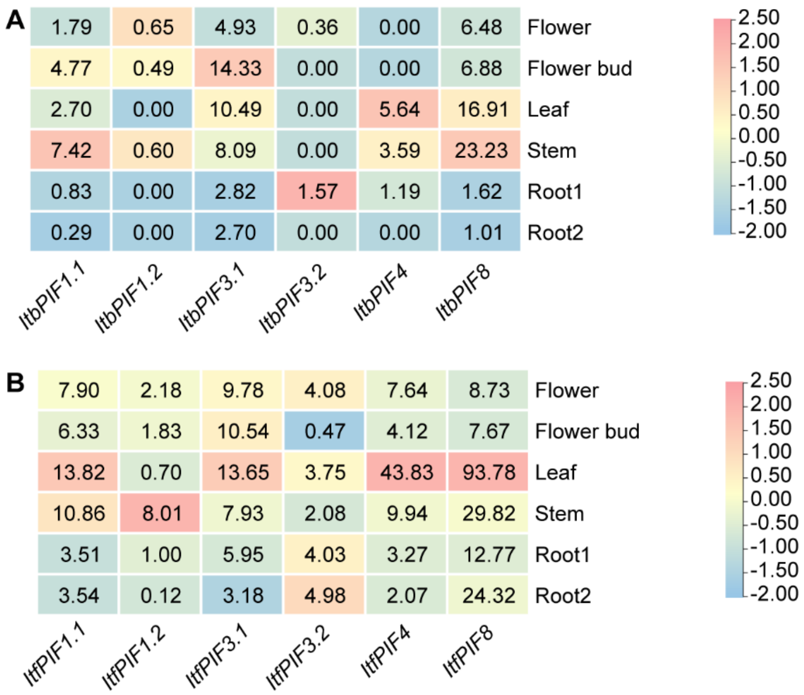

Figure S3. Gene expression patterns of (A) *ItbPIFs* and (B) *ItfPIFs* in different tissues (flower, flower bud, leaf, stem, root 1, and root 2) of *I. triloba* and *I. trifida*, as determined by RNA-seq. Log_2(FPKM + 1) is shown in the boxes. The red color denotes high expression and blue low expression.

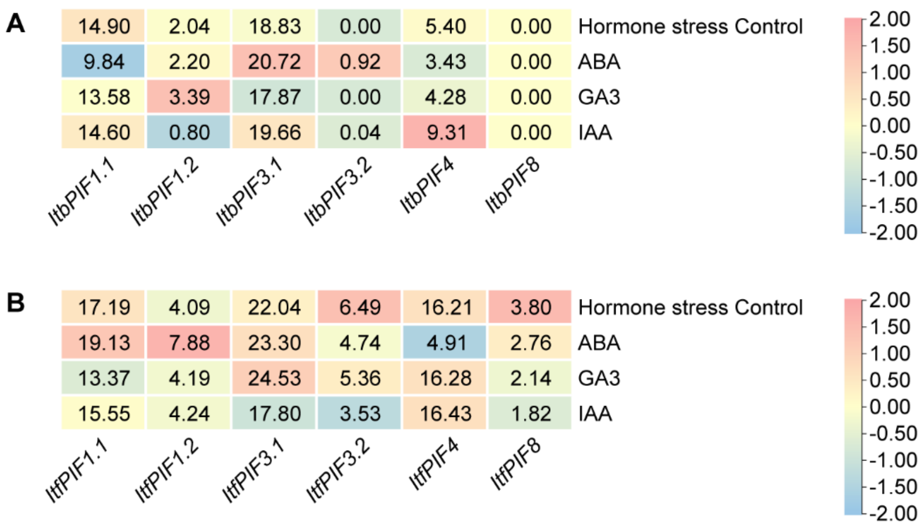

Figure S4. Gene expression patterns of (A) *ItbPIFs* and (B) *ItfPIFs* in response to different hormone (ABA, GA3, and IAA) of *I. triloba* and *I. trifida*, as determined by RNA-seq. Log_2(FPKM + 1) is shown in the boxes. The red color denotes high expression and blue low expression.

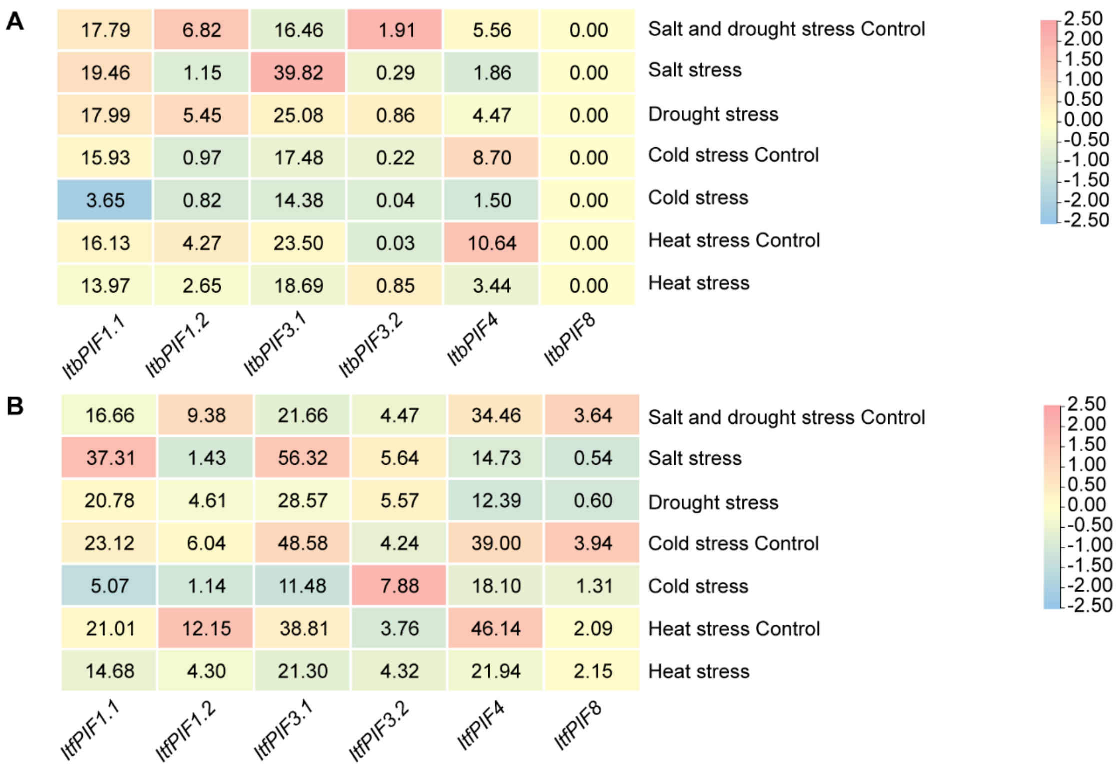

Figure S5. Gene expression patterns of (A) *ItbPIFs* and (B) *ItfPIFs* under abiotic stresses (salt, drought, cold, and heat) of *I. triloba* and *I. trifida*, as determined by RNA-seq. Log_2(FPKM + 1) is shown in the boxes. The red color denotes high expression and blue low expression.

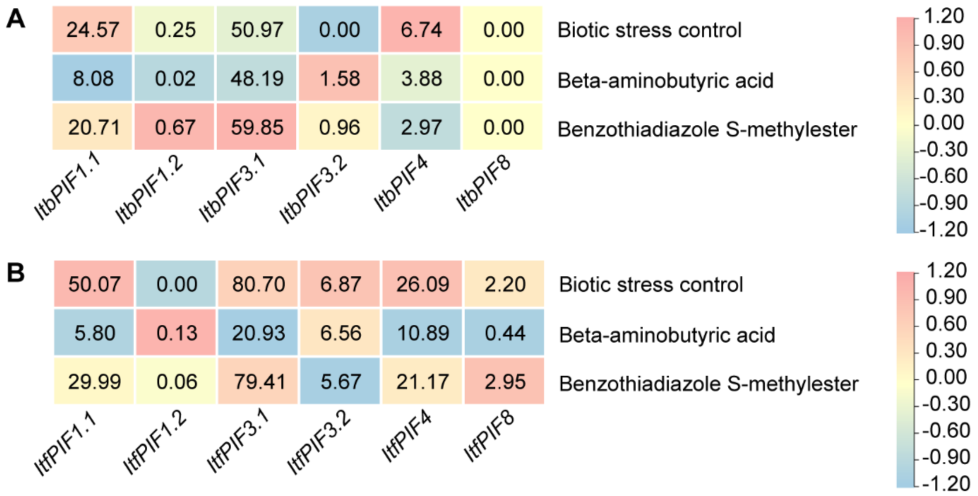

Figure S6. Gene expression patterns of *ItbPIFs* (A) and *ItfPIFs* (B) under biotic stresses (beta-aminobutyric acid biotic stress and benzothiadiazole S-methylester biotic stress) of *I. triloba* and *I. trifida*, as determined by RNA-seq. Log_2(FPKM + 1) is shown in the boxes. The red color denotes high expression and blue low expression.
